# Use of Dexmedetomidine With Dexamethasone for Extended Pain Relief in Adductor Canal/Popliteal Nerve Block During Achilles Tendon Repair

**DOI:** 10.7759/cureus.11917

**Published:** 2020-12-05

**Authors:** Hisham Kassem, Ivan Urits, Omar Viswanath, Alan D Kaye, Jonathan P Eskander

**Affiliations:** 1 Anesthesiology and Perioperative Medicine, Mount Sinai Medical Center, Miami Beach, USA; 2 Anesthesia, Critical Care and Pain Medicine, Beth Israel Deaconess Medical Center, Harvard Medical School, Boston, USA; 3 Pain Management, Valley Anesthesiology and Pain Consultants, Phoenix, USA; 4 Anesthesiology, Louisiana State University Health Sciences Center, Shreveport, USA; 5 Anesthesiology and Pain Medicine, Portsmouth Anesthesia Associates, Portsmouth, USA

**Keywords:** regional anesthesia, opioid-free analgesia, dexmedetomidine, dexamethasone, adductor canal block, popliteal nerve block

## Abstract

The use of regional anesthetic techniques in the peri-operative period has increased as a means to improve analgesia and patient outcomes. Traditionally, various local anesthetics are used and addition of adjuncts such as epinephrine, clonidine, and dexamethasone has shown to prolong the block effect. There has been ongoing research to support the prolongation of a regional block with the addition of dexamethasone and dexmedetomidine (Dex-Dex), providing analgesia for multiple days. We present a case of a 35-year-old female who underwent an Achilles tendon repair with an adductor/popliteal nerve block. Both dexmedetomidine and dexamethasone were added to the local anesthetic mixture with substantial postoperative analgesic control. The patient also did not require any supplemental opioid medication. This case emphasizes the proposed synergistic effect of dexmedetomidine and dexamethasone when added to the local anesthesia injectate for lower extremity peripheral nerve blocks.

## Introduction

Epinephrine, clonidine, and dexamethasone are used to extend peripheral nerve block duration [1-3]. Dexmedetomidine has shown promise as an adjunct in regional anesthesia [4]. When added to local anesthetic mixtures for regional anesthesia, it enhances its positive benefits [5, 6]. However, the synergistic effects of adding both dexamethasone and dexmedetomidine (Dex-Dex) to the block have yet to be properly studied.

## Case presentation

We present a 35-year-old 50-kg female who presented for an Achilles tendon repair following rupture. Informed consent was provided by the patient. Prior to the induction of general anesthesia, she underwent both an adductor canal and a popliteal nerve block for surgical and postoperative analgesia.

The patient was placed in the supine position, with the thigh abducted and externally rotated. Under sterile conditions and ultrasound guidance, a high-frequency linear transducer was placed anteromedially between the middle and distal third of the thigh to identify the femoral artery. Once identified, the artery was traced distally until it passed through the adductor hiatus. A 4-cm 21-gauge block needle was inserted via an in-plane technique and advanced through the sartorius muscle in the direction of the artery. After negative aspiration, a 10-mL mixture of 0.2% ropivacaine with 5 mg preservative-free dexamethasone and 25 mcg of dexmedetomidine were injected into the canal space.

Subsequently, the patient was placed in the lateral position with the foot elevated to optimize exposure, and a popliteal block was performed. Under ultrasound guidance, the transducer was placed transversely at the popliteal crease, and the popliteal artery identified. The transducer was then used to scan for the sciatic nerve just at the bifurcation of the tibial and common peroneal nerves. A 4-cm 21-gauge block needle was inserted via an in-plane technique in a horizontal orientation from the lateral aspect of the thigh and advanced toward the sciatic nerve. With negative aspiration, a 20-mL mixture of 0.2% ropivacaine with 5 mg preservative-free dexamethasone and 25 mcg of dexmedetomidine were injected. The patient tolerated both procedures well with no noted complications. Impressively, the patient reported a numerical rating scale pain score of 0/10 at rest for seven days after the surgery and had the onset of 2/10 pain eight days after. Of note, the patient did report residual sensory blockade at the anterior aspect of her ankle, which persisted until the 15th postoperative day. Motor blockade resolved on postoperative day 1 after a little more than 24 hours. Sensory blockade resulting from the adductor canal block resolved around postoperative day 3. In our experience, this duration is more typical of the dex-dex combination nerve blocks [7-10]. The popliteal fossa block duration, in this case, is unusually long. This may be attributed to sensitivity of the sciatic nerve at the popliteal fossa to this combination of local anesthetic plus adjuncts or could be related to neuropraxia. Of note, this patient did not have any pain either at rest or when she moved her leg. She also noted that even though she was not supposed to bear weight on her operative leg this still did not produce discomfort. Remarkably, the patient did not use any opioid or non-opioid analgesic medications postoperatively.

## Discussion

While the synergistic benefit of dexmedetomidine with dexamethasone in mixtures for peripheral nerve blocks may offer improved analgesic effect, there are inherent risks associated with their use. The risk of intravascular injection or prolonged absorption may lead to systemic side effects, including bradycardia, hypotension, and sedation [11]. The ideal dose of additive medications has also yet to be confirmed. A study by Marhofer and Brummett noted that the ideal balance between optimization of block strength and potentiation of side effects was at a dose of 100 mcg of dexmedetomidine [12]. Since this was with the use of dexmedetomidine alone and significantly lower doses may be needed with the inclusion of dexamethasone.

In summary, the use of Dex-Dex with local anesthetics for peripheral nerve blocks during the perioperative period can provide a greater reduction of pain while minimizing opioid use. Moreover, the theoretical risk of local anesthetic toxicity is reduced when additives are used, but the ideal combination of medications has yet to be reported. With more improved pain control, this can ultimately lead to decreased length of hospital stay and a faster return of a patient’s normal level of functioning.

## Conclusions

While this case is a single experience, we are confident that further investigations can strengthen the notion that the addition of both dexamethasone with dexmedetomidine to local anesthetic mixtures for peripheral nerve blocks is safe and efficacious. However, given the higher likelihood of nerve injury to the sciatic nerve at the site of the popliteal fossa, we cannot recommend the use of the dex-dex combination without further investigation.
